# Development of flow cytometry assay to quantify packaging of *C. jejuni* by *Tetrahymena* species

**DOI:** 10.1093/femsle/fnaf114

**Published:** 2025-10-17

**Authors:** Madison Schacter, Sara Matthews, Valérie E Paquet, Hana Trigui, Jennifer Ronholm, Steve J Charette, Sebastien P Faucher

**Affiliations:** Department of Natural Resource Sciences, McGill University, Saint-Anne-de-Bellevue, QC H9X3V9, Canada; Centre de Recherche et Infectiologie Porcine et Avicole (CRIPA), Université de Montréal, Faculté de Médécine Vétérinaire, Saint-Hyacinthe, QC J2S 2M2, Canada; Department of Natural Resource Sciences, McGill University, Saint-Anne-de-Bellevue, QC H9X3V9, Canada; Centre de Recherche et Infectiologie Porcine et Avicole (CRIPA), Université de Montréal, Faculté de Médécine Vétérinaire, Saint-Hyacinthe, QC J2S 2M2, Canada; Institut de Biologie Intégrative et des Systèmes (IBIS), Pavillon Charles-Eugène-Marchand, Université Laval, Québec City, QC G1V 0A6, Canada; Département de biochimie, de microbiologie et de bio-informatique, Faculté des sciences et de génie, Université Laval, Québec City, QC G1V 0A6, Canada; Department of Natural Resource Sciences, McGill University, Saint-Anne-de-Bellevue, QC H9X3V9, Canada; Centre de Recherche et Infectiologie Porcine et Avicole (CRIPA), Université de Montréal, Faculté de Médécine Vétérinaire, Saint-Hyacinthe, QC J2S 2M2, Canada; Department of Food Sciences, McGill University, Saint-Anne-de-Bellevue, QC H9X3V9, Canada; Centre de Recherche et Infectiologie Porcine et Avicole (CRIPA), Université de Montréal, Faculté de Médécine Vétérinaire, Saint-Hyacinthe, QC J2S 2M2, Canada; Institut de Biologie Intégrative et des Systèmes (IBIS), Pavillon Charles-Eugène-Marchand, Université Laval, Québec City, QC G1V 0A6, Canada; Département de biochimie, de microbiologie et de bio-informatique, Faculté des sciences et de génie, Université Laval, Québec City, QC G1V 0A6, Canada; Department of Natural Resource Sciences, McGill University, Saint-Anne-de-Bellevue, QC H9X3V9, Canada; Centre de Recherche et Infectiologie Porcine et Avicole (CRIPA), Université de Montréal, Faculté de Médécine Vétérinaire, Saint-Hyacinthe, QC J2S 2M2, Canada; Centreau – Centre québécois de recherche sur la gestion de l’eau, Université Laval, Québec City, QC G1V 0A6, Canada

**Keywords:** multilamellar bodies, bacteria packaging, flow cytometry, *Campylobacter jejuni*, *Tetrahymena*

## Abstract

The human pathogen *Campylobacter jejuni* can be packaged within multilamellar bodies (MLB), also called fecal pellets, produced by ciliates such as *Tetrahymena pyriformis* when these microorganisms are cocultivated. This packaging increases the survival of *C. jejuni* in oxygenic conditions and potentially protects it against other stressors. Traditional methods for detecting and quantifying these pellets, such as transmission electron microscopy (TEM) and fluorescence microscopy, are time-consuming and labor intensive. In this study, we devised an approach for utilizing flow cytometry to distinguish and quantify *C. jejuni*-containing pellets produced by both *T. pyriformis* and *T. thermophila*. Cocultures of each *Tetrahymena* species with four different *C. jejuni* strains, along with monoculture controls, were incubated for 24 h, stained with SYTO9, and analysed using flow cytometry. The results revealed ciliate species-specific and bacterial strain-specific differences in the number of pellets and their fluorescence intensity. TEM confirmed that this variability in fluorescence corresponds to differences in the number of bacteria per pellet. Our method provides a rapid and efficient means of quantifying bacteria-containing MLBs, which would facilitate the screening and comparison of a large quantity of *C. jejuni* strains and different conditions for studying the packaging of *C. jejuni* by ciliates.

## Teaser

A flow cytometry method was developed to measure more accurately the packaging of the water-borne pathogen *Campylobacter* by the ciliate *Tetrahymena*, which might promote its transmission by water.

## Introduction

Protozoa are unicellular eukaryotes that are ubiquitous in many naturally occurring and engineered environments, such as rivers and lakes, water distribution systems, and evaporative cooling towers, where they play significant ecological roles (Durocher et al. 2020). Many protozoa are grazers that feed by ingesting other microorganisms, like bacteria (Denoncourt et al. 2014). Typically, when ciliates prey on bacteria, the bacteria are captured through phagocytosis, where they are engulfed and transferred to lysosomal compartments for enzymatic digestion. Protozoa and bacteria have coexisted and interacted for a long time, leading some bacteria, such as *Legionella pneumophila* and *Mycobacterium* spp., to evolve mechanisms that enable them to use protozoa as hosts (Greub and Raoult 2004). Among these adaptations, they can evade the digestion process (Richards et al. 2013). For example, *L. pneumophila* induces the formation of a replication vacuole inside of protozoa, thereby blocking maturation of the phagolysosome and allowing its survival and growth inside the protozoan host (Richards et al. 2013).

Multilamellar bodies (MLBs) are lysosome-derived structures characterized by multiple concentric membrane layers (Schmitz and Müller 1991). Many protozoa, including ciliates and amoebae, regularly expel these structures as fecal pellets as part of their normal digestive process (Paquet et al. 2013). These fecal pellets often contain undigested particulates and other organic nutrients (Buck et al. 1990). In some cases, bacteria are among the materials packaged within MLBs and are expelled into the environment still alive (Denoncourt et al. 2014). Thus, MLBs can serve as protective vehicles for viable bacteria, allowing their release from protozoa without digestion. Packaging within MLBs offers several advantages to bacteria, including increased resistance to antibiotics, biocides, low pHs, and other environmental stressors (Berk et al. 2008). It can also enhance the survival of bacteria when exposed to long-term starvation (Espinoza-Vergara et al. 2019). Several bacteria, pathogenic or not, can be packaged by protozoa, including, *Mycobacterium smegmatis, L. pneumophila, Salmonella enterica*, and *Campylobacter jejuni* (Berk et al. 2008, Gourabathini et al. 2008, Trigui et al. 2016, Denoncourt et al. 2017, Durocher et al. 2020).

*Campylobacter jejuni*, a human pathogen, is a leading global cause of gastroenteritis, with symptoms including high fever and watery diarrhea. In more extreme cases, symptoms can include bloody diarrhea, nausea, and vomiting (Blaser and Engberg 2008). Humans can consume and be infected with *C. jejuni* through ingestion of contaminated food and water. According to Vrbova et al. (2012), *Campylobacter* accounted for 36.5% of all reported gastrointestinal illnesses in Ontario (Canada) from 2007 to 2009. Among the cases with primary sources, about 63.1% were attributed to contaminated food, and 2.8% were attributed to contaminated water, including drinking and recreational water (Vrbova et al. 2012).

*Campylobacter jejuni* is a microaerophilic bacterium normally found in chickens and livestock, where they are released in large numbers within feces, which contaminate the environment (Levesque et al. 2013). Fecal contamination of private wells and surface water can then occur via rainwater runoff, particularly during flood conditions, or through the application of manure to farmland (Levesque et al. 2013). In rural areas, consumption of untreated or improperly disinfected private well water significantly increases the risk of infection by *C. jejuni* (Levesque et al. 2013). Water isolates of *C. jejuni* have been found to belong to clonal complexes also present in human infections, highlighting water as a potential source of human exposure (Levesque et al. 2013).

In 2005, Snelling et al. (2005) coisolated *Campylobacter* and protozoa from the same drinking water system, indicating a potential interaction between the two microorganisms. Bui et al. (2012) later demonstrated that *Acanthamoeba castellanii* enhances the growth of *C. jejuni* primarily by depleting dissolved oxygen even without physical interaction between amoeba and bacteria. In 2016, packaging of *C. jejuni* strain 81116 into MLBs by the ciliate *Tetrahymena pyriformis* was shown (Trigui et al. 2016). Packaged *C. jejuni* survived longer in aerobic condition than free *C. jejuni* indicating a potential benefit for transmission between animal hosts. Given the cooccurrence of ciliates and *C. jejuni* in aquatic environments, it is plausible that packaging of *C. jejuni* by ciliates could enhance its survival and dissemination in water.

Detection and quantification of fecal pellet production have traditionally been carried out using imaging techniques such as transmission electron microscopy (TEM) and epifluorescent microscopy (Paquet and Charette 2016, Trigui et al. 2016, Durocher et al. 2020). While these methods can provide detailed images, they are often labor-intensive and time-consuming, because accurate quantification requires a manual counting of many images, particularly at lower concentrations. Consequently, there is a need for a faster method, especially if pellet formation is to be used as a screening tool for bacterial strain characterization.

Flow cytometry offers a promising alternative due to its ability to rapidly analyse individual cells or particles, ranging in size from 0.2 to 150 µm, as they pass through one or more laser beams (Davey and Kell 1996). By measuring forward and side-scattered light, flow cytometry can differentiate between populations based on size and granularity, or internal complexity. Fluorescence-based detection further enhances the capability of distinguishing specific populations through either inherent fluorescence or the use of fluorescent markers. This technology has become widely adopted in microbiology, allowing for the rapid and quantitative assessment of cell populations, their physiological states, and viability (McKinnon 2018).

In this study, we developed a method to detect and quantify fecal pellets containing *C. jejuni*, produced by *Tetrahymena* using flow cytometry. Furthermore, we compared the packaging of four bacterial strains, two human clinical isolates and two water isolates, in coculture with two species of ciliates, *T. pyriformis* and *Tetrahymena thermophila*.

## Materials and methods

### Ciliates and *C. jejuni* strains

*Campylobacter jejuni* strains used in this study include 81116 (Pearson et al. 2007) and NCTC11168 isolated from human clinical cases (Parkhill et al. 2000) as well as 001B-0359 isolated from chicken and 007A-1431 isolated from a water sample in Coaticook, Québec (Lévesque et al. 2013). Prior to each experiment, bacteria were grown at 42°C for 72 h on blood agar plates, prepared from tryptic soy agar (Difco) and 5% (v/v) defibrinated sheep blood (Cedarlane, Canada), in a microaerobic environment generated with a CampyGen gas-generating system (ThermoScientific, United Kingdom).

*Tetrahymena pyriformis* ATCC 30202 and *T. thermophila* ATCC 16539 were obtained from Cedarlane, Canada. *Tetrahymena pyriformis* and *T. thermophila* were routinely grown axenically at 21°C in ATCC Medium 327 (5 g/l proteose peptone, 5 g/l tryptone, and 0.2 g/l K_2_HPO_4_) in cell culture flasks (75 cm^2^; Sarstedt). To expand culture, 3 days prior to the experiment, 1 ml of culture was passaged into 50 ml of sugar–protease–peptone (SPP) (10 g/l proteose peptone, 1 g/l dextrose, 0.5 g/l yeast extract, and 0.033 mM FeCl_3_) and grown at 21°C.

### *Campylobacter jejuni*–ciliate cocultures

The bacterial lawn was harvested from blood agar medium and gently resuspended in 1 ml plate count broth (PCB) (5 g/l yeast extract, 10 g/l tryptone, and 2 g/l dextrose). The bacterial suspension was diluted to an OD_595_ of 0.1 in 6.5 ml PCB, resulting in ∼1 × 10^8^ CFU/ml. Separately, 15 ml of ciliate culture in SPP medium was centrifuged a 600 × *g* for 2 min. The supernatant was discarded, and the pellet resuspended in 5 ml of PCB. Hemocytometer counts were performed, and the cells were then diluted with PCB to a final concentration of 5 × 10^5^ cells/ml. In T25 plastic culture flasks (Greiner Bio-One, Monroe, NC, USA), *Tetrahymena* ciliates were inoculated at 9 × 10^4^ cells/ml, and *C. jejuni* at 2 × 10^7^ CFU/ml in PCB. Axenic cultures of ciliates or bacteria were also prepared as controls. All cocultures and controls were prepared in triplicate and incubated at room temperature (21°C) for 24 h before being analysed by flow cytometry. Each experiment was performed three times.

### Flow cytometry measurements

**Figure 1.**
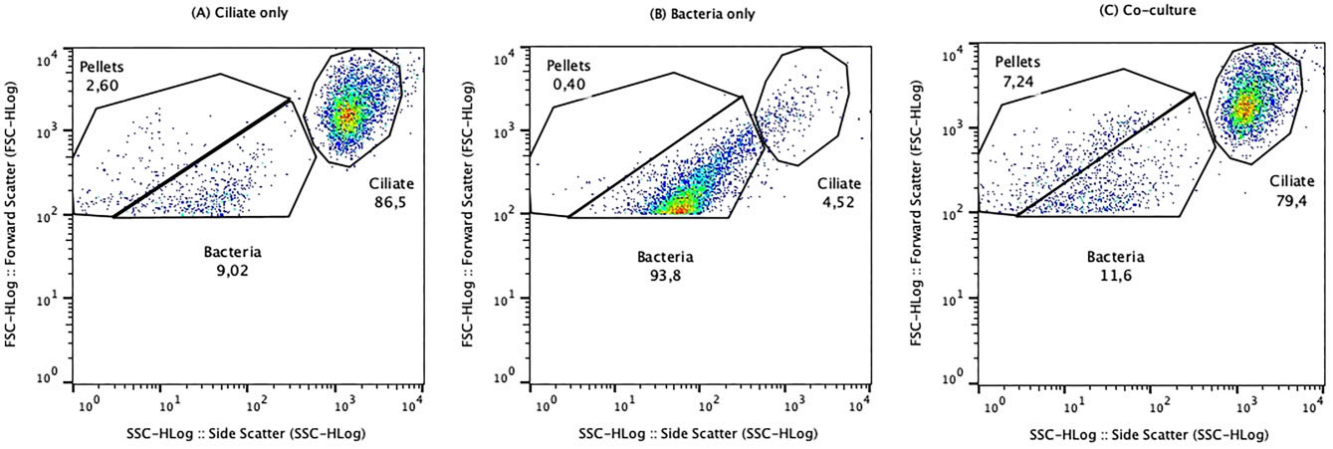
Example of flow cytometry FSC versus SSC plots for (A) monoculture of *T. pyriformis*, (B) monoculture of *C. jejuni* NCTC11168, and (C) coculture of *T. pyriformis* and NCTC11168. Gates are drawn around bacteria, ciliate, and pellet populations based on monoculture controls. Threshold was set to 100. Plots were generated with FlowJo software.

**Figure 2.**
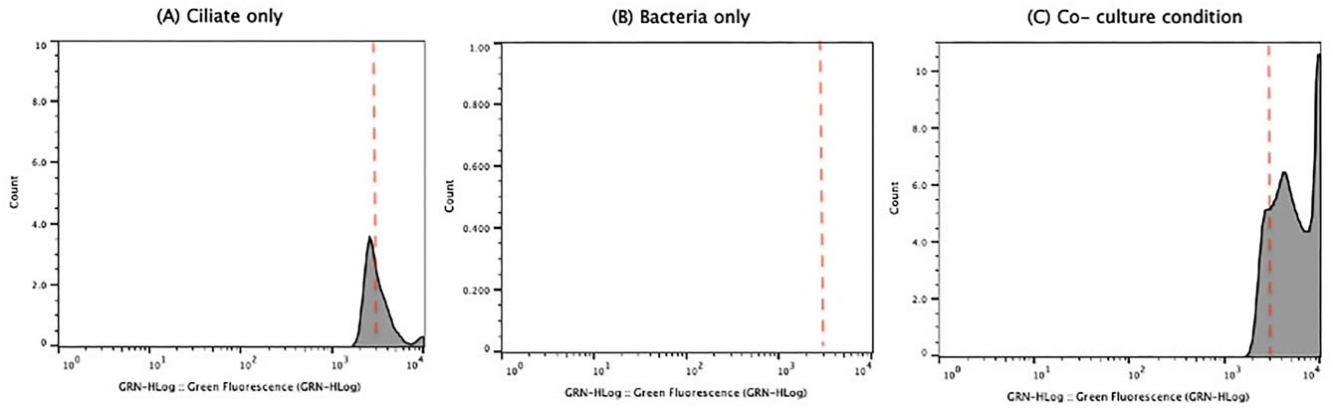
Histogram of fluorescence intensity from green fluorescence versus event count plots. Flow cytometry histograms display green fluorescence (GRN-HLog) for (A) *T. pyriformis* species only, (B) *C. jejuni* NCTC11168 only, and (C) coculture of *T. pyriformis* and *C. jejuni* NCTC11168. Events showing fluorescence value superior to the broken line were considered to have high fluorescence. Histograms were generated with FlowJo software.

After the 24 h incubation, pellet formation was assessed using flow cytometry. From each culture containing either axenic *Tetrahymena*, axenic *C. jejuni*, or ciliate–bacteria coculture, two 500 µl aliquots were mixed with 250 µl of 4% formaldehyde in phosphat-buffered saline (PBS) to fix cells, and then immediately centrifuged at 3000 × *g* for 3 min. The supernatant was discarded, and the pellet was resuspended in fresh 500 µl of 4% formaldehyde in PCB. One aliquot from each of the flasks was stained with SYTO9, gently mixed, and incubated in the dark for 15 min at room temperature. Then, samples were centrifuged again at 3000 × *g* for 3 min, the supernatant was discarded, and pellets were resuspended in fresh 500 µl of 4% formaldehyde in PBS. Measurements of the forward (FSC) and side scatter (SSC) and green fluorescence were taken with a Guava easyCyte (Millipore). Gains were adjusted to visualize the ciliate and bacterial populations, based on ciliate and bacteria alone controls. Noise was minimized at the beginning of each experiment by setting a FSC threshold of 100. Populations were gated to ensure the analysis was focused on events that represent intact cells or particles of interest. For analysis, a gate was defined based on FSC and SSC plots of the ciliate, bacteria, and PBS only controls (Fig. 1). The pellet population was defined as the one that had intermediate forward scattering intensity, as the pellet size is between that of the bacteria and ciliates, and this population only appeared in small quantities in ciliate-only controls, and large quantities in ciliate–bacterial cocultures and was absent in bacteria only controls. To identify pellet events that contained internalized fluorescent bacteria, Green Fluorescence versus Event Count histograms were generated from the gated pellet population. A fluorescence threshold was established based on the maximum fluorescence intensity observed in the ciliate-only control. (Fig. 2). Events in the coculture samples that exceeded this threshold were considered to represent pellets containing SYTO9-stained bacteria. This approach allowed us to distinguish between empty pellets and those likely filled with bacteria based on fluorescence intensity above background levels.

### TEM

To prepare samples for TEM, cocultures of *C. jejuni* with *T. pyriformis* were fixed for 3 h in a 0.1 M sodium cacodylate buffer (pH 7.3) containing 2% glutaraldehyde and 0.3% osmium tetroxide. After fixation, samples were embedded in Epon resin and sectioned into ultrathin slices according to standard electron microscopy procedures as previously described by Paquet et al. (2013). Imaging was performed using a JEOL 1230 TEM operating at 80 kV at the imaging and microscopy platform of Institut de Biologie Intégrative et des Systèmes (IBIS).

## Results

### Distinguishing fecal pellets using flow cytometry

To determine whether bacteria, ciliates, and fecal pellets could be differentiated using flow cytometry, we analysed monocultures of bacteria and ciliates as controls, along with cocultures (Figs 1 and 2). The ciliate population (Fig. 1A) appeared as events with higher forward scatter (FSC-H) and side scatter (SSC-H) compared to the bacteria population (Fig. 1B), reflecting the smaller size of bacteria relative to ciliates. In the coculture samples (Fig. 1C), a distinct population appeared in the FSC versus SSC plots, which was absent in the bacteria only controls and present in minor quantities in the ciliate only controls. This population, characterized by intermediate scatter values, likely corresponds to pellets produced by *Tetrahymena*, which are more abundant during coculture with *C. jejuni* (Trigui et al. 2016). These 2–5 µm pellets exhibit distinct internal complexity and occupy a size range between that of ciliates (∼40–60 µm) and bacterial particles (∼1 µm) (Trigui et al. 2016).

### Fluorescence analysis of pellet events

A previous study indicated that *C. jejuni* cells packaged in pellets can be fully labeled with SYTO9 (Trigui et al. 2016). Building on this finding, we refined our analysis to better detect the presence of *C. jejuni* within the pellet population. To evaluate fluorescence within the pellet population, we focused on the events gated as pellets and generated histograms displaying green fluorescence (GRN-HLog) versus events count (Fig. 2). These histograms allowed us to quantify the number of highly fluorescent pellets, defined as those exhibiting fluorescence above that of the ciliate-only control. The ciliate-only condition had a low amount of high fluorescence pellets (Fig. 2A), and the bacteria-only condition displayed little-to-no highly fluorescent events (Fig. 2B). The histograms show that the greatest number of highly fluorescent pellets is observed in the coculture conditions (Fig. 2C). This pattern suggests that the presence of fluorescent *C. jejuni* in a population of ciliates contributes to the formation of fluorescent pellets, and that the ciliate–bacterial interaction in coculture further enhances the number of high fluorescence pellets, likely due to the packaging of fluorescent *C. jejuni* within the pellets.

### Quantification of pellets by flow cytometry

**Figure 3.**
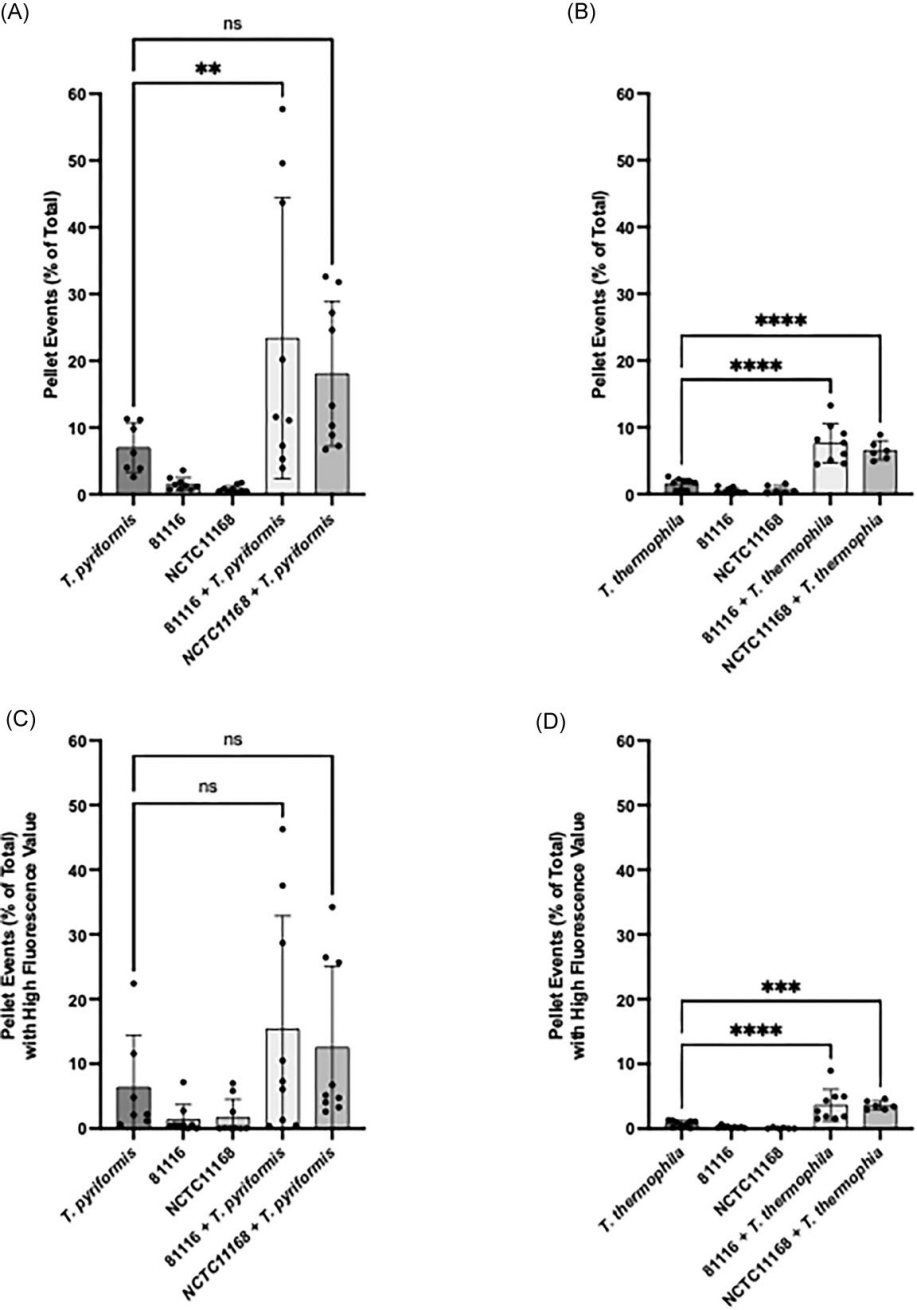
Quantification of pellet events and their fluorescence levels in *T. pyriformis* and *T. thermophila* cocultures with *C. jejuni* strains 81116 and NCTC11168. (A and B) Pellet events are expressed as a percentage of the total number of recorded events for each condition. (C and D) Pellet events with high fluorescence values were quantified to assess bacteria packaging. The data represent three experiments each performed in triplicates. Statistical significance was determined using a one-way ANOVA, with multiple comparisons performed using a *t*-test without correction (* is *P* < .05, ** is *P* < .01, *** *P* < .001, and **** *P* < .0001).

To evaluate the impact of *C. jejuni* strains on pellet formation, *T. pyriformis* and *T. thermophila* were cocultured with the human clinical isolates 81116 and NCTC11168, and the percentage of pellet events relative to total recorded events was measured (Fig. 3A and B). The strain 81116 serve as positive control, as it was previously shown to be packaged effectively by *T. pyriformis* (Trigui et al. 2016). The results showed that both ciliates produced an increased number of pellets in the presence of strain 81116 compared to the bacteria-free controls (Fig. 3A and B). A significant increase in the number of pellet production by *T. thermophila* was observed when cocultured with NCTC11168 (*P* < .0001, Fig. 3B), whereas no such significative increase was detected for *T. pyriformis* (Fig. 3A).

Further analysis using the fluorescence intensity within the pellet population revealed that high-fluorescence pellet formation was increased significantly by *T. thermophila* for 81116 (*P* < .0001) and NCTC11168 (*P* < .001). This suggests that, in addition to inducing pellet formation, both strains are efficiently packaged into pellets produced by the species *T. thermophila*. However, in *T. pyriformis*, neither strain of *C. jejuni* increased the number of high-fluorescent pellets significantly.

**Figure 4.**
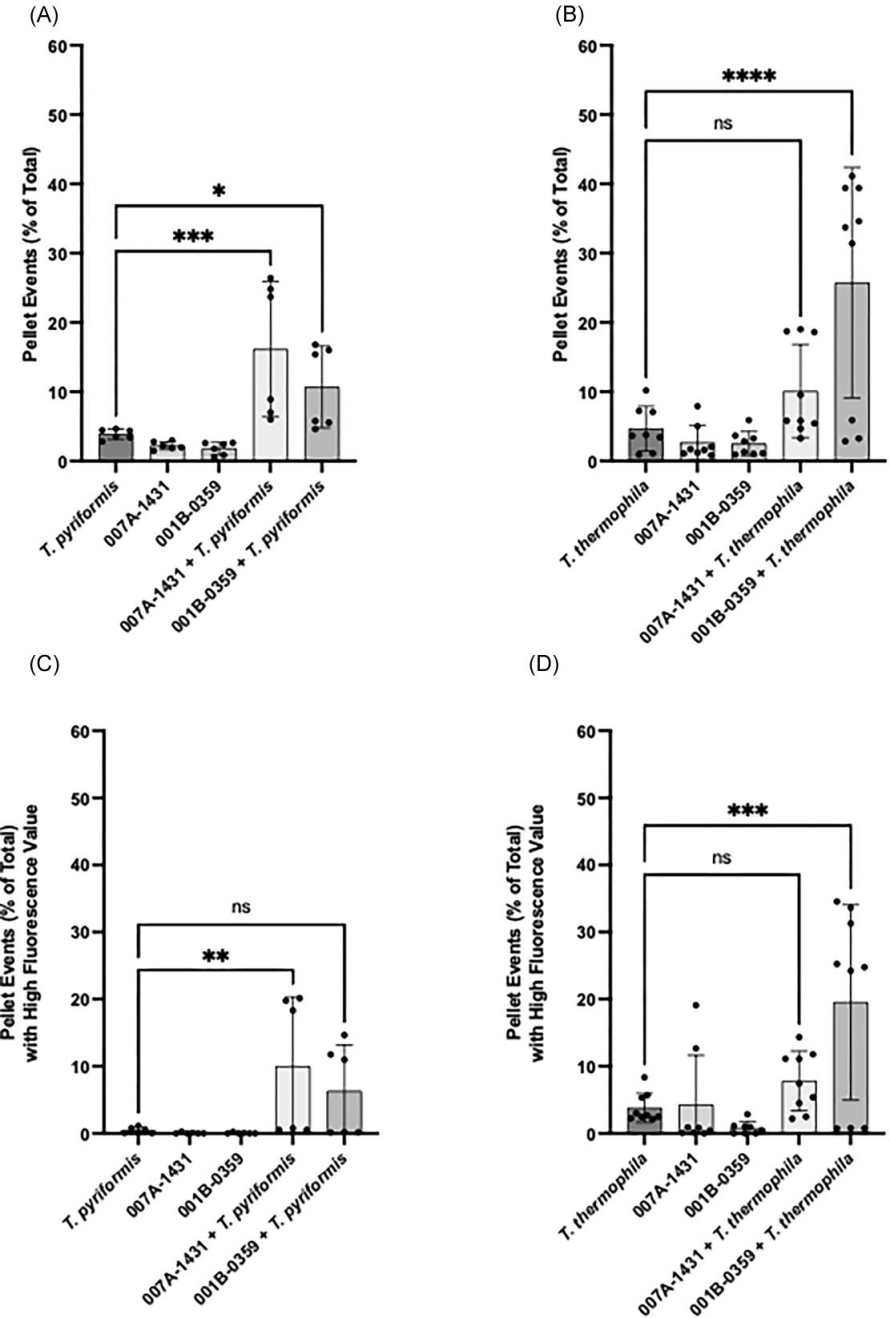
Quantification of pellet events and their fluorescence levels in *T. pyriformis* and *T. thermophila* cocultures with *C. jejuni* strains 007A-1431 and 001B-0359. (A and B) Pellet events are expressed as a percentage of the total number of recorded events for each condition. (C and D) Pellet events with high fluorescence values were quantified to assess bacterial uptake. The data represent three experiments each performed in triplicates. Statistical significance was determined using a one-way ANOVA, with multiple comparisons performed using a *t*-test without correction (* is *P* < .05, ** is *P* < .01, *** *P* < .0001, and **** *P* < .0001).

To assess whether nonhuman isolates interact differently with ciliates compared to clinical strains, *T. pyriformis* and *T. thermophila* were cocultured with 007A-1431 and 001B-0359. In cocultures with *T. pyriformis*, both strains of *C. jejuni* led to an increase in overall pellet production compared to monoculture controls, with 007A-1431 being associated with a greater pellet production (*P* < .001) but not 001B-0359 (*P* > .05). For coculture conditions with *T. thermophila*, only interactions with 001B-0359 cause a significant increase in pellet production (*P* < .0001, Fig. 4B). When assessing the bacteria packaging into pellets using the number of high-fluorescing pellets, the interaction with 007A-1431 and *T. pyriformis* (*P* < .01, Fig. 4C) and the interaction with 001B-0359 and *T. thermophila* (*P* < .001, Fig. 4D) led to the efficient packaging of the bacteria into the pellet.

**Figure 5.**
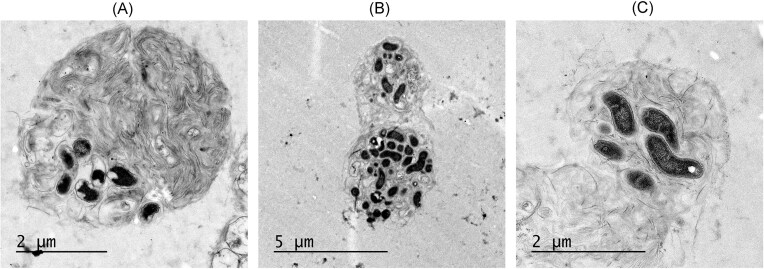
TEM of *C. jejuni* strain 81116 packaged into pellets by *T. pyriformis*. Panels show a variety of pellets with a variable number of bacteria and morphology. The black spots represent *C. jejuni*, while the surrounding gray material is the protein matrix produced by the ciliate.

The variability in the fluorescence intensity of the pellets suggests that the packaging of *C. jejuni* in pellets may not be uniform. This was investigated by TEM of *C. jejuni* strain 81116 packaged by *T. pyriformis*. Panels A–C in Fig. 5 illustrate the varying levels of bacterial abundance within the pellets, confirming the fluorescence intensity variability observed by flow cytometry for pellets produced by *T. pyriformis*.

## Discussion

This study demonstrates that flow cytometry combined with SYTO9 staining can be used to quantify bacteria-containing pellets produced by *Tetrahymena* species in coculture with *C. jejuni*. By analysing FSC and SSC signals and then the fluorescence signal, we were able to distinguish bacteria, ciliates and fecal pellets, providing a rapid and efficient alternative to microscope-based methods. The characteristics of bacteria, ciliate, and pellet populations seem to agree with the size of the pellets (∼2–5 µm), the ciliates (∼40–60 µm) and the bacteria populations (∼1 µm) (Trigui et al. 2016). While a previous study by First et al. (2012) used flow cytometry to monitor the ingestion and digestion of fluorescently labeled *C. jejuni* by ciliates, their focus was on intracellular bacteria. In contrast, our method specifically quantifies expelled MLBs containing bacteria, offering a novel application of flow cytometry to study this distinctive packaging process.

In the first study on *C jejuni* packaging by ciliates, only a single bacterial strain and one ciliate species were tested (Trigui et al. 2016). In contrast, the present study, using a quantitative approach with multiple bacterial strains—both clinical and environmental isolates—and two ciliate species, provides new insights into this phenomenon. The findings demonstrate that pellet formation and bacterial packaging depend on both the bacterial strain and the ciliate species. Some *C. jejuni* strains were more effectively packaged than others. Significant variability in pellet formation was observed, both between different strains and within the same strain across experiments, which was confirmed with TEM. A previous study on bacteria packaging by amoeba *Dictyostelium discoideum* highlighted the substantial variability in the packaging process among strains of the same species (Paquet and Charette 2016). Another study investigating bacterial packaging by *T. thermophila* and *T. pyriformis* across various nonpathogenic bacterial species found that *T. pyriformis* produced bacteria-containing pellets with a greater diversity of bacteria and in higher amounts, based on a semiquantitative microscopic analysis (Durocher et al. 2020). In the present study, *T. thermophila* demonstrated superior statistically significant production of bacteria-filled fluorescent pellets for the clinical *C. jejuni* strains, and for one of the environmental strains.

One of the key challenges in this study is the variability in pellet formation and fluorescence intensity across experiments. This variability likely stemmed from biological factors related to both *Tetrahymena* and *C. jejuni*. The physiological state of the ciliates may have influenced pellet production as grazing efficiency and expulsion behavior is affected by how long the culture has been passaged (Durocher et al. 2020). Since ciliates expel fecal pellets as part of their normal digestive process (Denoncourt et al. 2014), the rate at which they form and expel pellets may fluctuate depending on their feeding activity and environmental conditions. The stage of bacterial growth at the time of coculture could also influence how effectively *C. jejuni* is internalized and expelled in pellets, as bacteria in different physiological states exhibit varying levels of resistance to protozoan digestion (Berk et al. 2008). Moreover, difficult-to-control variations in bacteria–ciliate ratios may also partly explain the variability observed between different experiments. In the study done by Trigui et al. (2016), a higher multiplicity of infection (MOI) was used to ensure that the bacterial pellets were more frequently and easily observed under the microscope. The high MOI increased the likelihood of bacterial incorporation into pellets, facilitating their detection and analysis in a microscopic setting. In contrast, our study utilized flow cytometry, which allows for detection and quantification of pellets even when the bacterial concentration is lower. Despite the observed variability, explained by the factors outlined above, the cytometry approach remains promising, offering greater sensitivity than microscopy and enabling more straightforward quantification. Moreover, the lower bacteria-to-ciliate ratio used in this study may more accurately reflect the conditions found in natural environments where these microorganisms coexist. The variability observed in pellet formation and fluorescence intensity underscores the complexity of protozoan–bacterial interactions and suggests that additional environmental and biological factors influence packaging efficiency. Low to moderate levels of fluorescence were occasionally detected in ciliate-only control. This may be due to residual extracellular DNA, dying or autolyzing ciliate cells, or nonspecific SYTO9 uptake by organic debris in the culture medium. Some of these events may also represent empty fecal pellets, as ciliates naturally produce fecal pellets as part of their digestive process, even in axenic conditions (Denoncourt et al. 2014). Some fluorescence may result from endogenous nucleic acid-containing material released during normal metabolism. These background events were accounted for by setting a fluorescence threshold based on the ciliate-only controls. Future study should explore the effect of ciliate culture age, bacterial growth phase media composition, environmental stressors, on the size of pellet, and density of bacteria package in them.

Although SYTO9 staining provided a practical means to assess bacterial packaging within pellets, our findings suggest that future studies could benefit from alternative fluorescence-based strategies. For instance, the use of genetically modified *C. jejuni* strains expressing a fluorescent protein would offer a more direct and specific way to label bacterial cells. This approach could reduce background fluorescence caused by extracellular DNA or cellular debris, thereby improving the accuracy and specificity of quantification. Such a strategy represents a future avenue to refine the detection of bacteria-filled pellets in ciliate cocultures.

In conclusion, this study establishes flow cytometry as a useful method for quantifying bacterial packaging by *Tetrahymena*. Despite some variability in pellet formation and fluorescence that may affect direct comparisons across experiments, the consistent trends observed strongly support that packaging is determined by both the ciliate species and the bacterial strain.

## Data Availability

Data analysed during this study are provided in full within the published article and its supplementary materials. Raw flow cytometry data is available upon reasonable request.
